# Effects of Local Gravity Compensation on Motor Control During Altered Environmental Gravity

**DOI:** 10.3389/fncir.2021.750267

**Published:** 2021-10-21

**Authors:** Tjasa Kunavar, Marko Jamšek, Marie Barbiero, Gunnar Blohm, Daichi Nozaki, Charalambos Papaxanthis, Olivier White, Jan Babič

**Affiliations:** ^1^Laboratory for Neuromechanics and Biorobotics, Department of Automatics, Biocybernetics and Robotics, Jožef Stefan Institute, Ljubljana, Slovenia; ^2^Jožef Stefan International Postgraduate School, Ljubljana, Slovenia; ^3^INSERM UMR1093-CAPS, Université Bourgogne Franche-Comté, UFR des Sciences du Sport, Dijon, France; ^4^Centre National d’Etudes Spatiales, Paris, France; ^5^Centre for Neuroscience Studies, Queen’s University, Kingston, ON, Canada; ^6^Division of Physical and Health Education, Graduate School of Education, The University of Tokyo, Tokyo, Japan

**Keywords:** motor assistance, gravitational effects, parabolic flight, motor control, microgravity, hypergravity

## Abstract

Our sensorimotor control is well adapted to normogravity environment encountered on Earth and any change in gravity significantly disturbs our movement. In order to produce appropriate motor commands for aimed arm movements such as pointing or reaching, environmental changes have to be taken into account. This adaptation is crucial when performing successful movements during microgravity and hypergravity conditions. To mitigate the effects of changing gravitational levels, such as the changed movement duration and decreased accuracy, we explored the possible beneficial effects of gravity compensation on movement. Local gravity compensation was achieved using a motorized robotic device capable of applying precise forces to the subject’s wrist that generated a normogravity equivalent torque at the shoulder joint during periods of microgravity and hypergravity. The efficiency of the local gravity compensation was assessed with an experiment in which participants performed a series of pointing movements toward the target on a screen during a parabolic flight. We compared movement duration, accuracy, movement trajectory, and muscle activations of movements during periods of microgravity and hypergravity with conditions when local gravity compensation was provided. The use of local gravity compensation at the arm mitigated the changes in movement duration, accuracy, and muscle activity. Our results suggest that the use of such an assistive device helps with movements during unfamiliar environmental gravity.

## Introduction

Our sensorimotor control is adapted for the Earth’s environment, where all movements are conditioned by the gravitational force (Fisk et al., 1993). This omnipresent force is taken into account by our central nervous system (CNS) during all motor actions. Exposure to altered gravity significantly disturbs our movements (Bock et al., 1992). Aimed arm movements, such as pointing and reaching, constitute complex acts of sensorimotor integration, and gravitational information is imperative when anticipating the consequences of motor commands on the position of the arm (Bock et al., 1992). Arm movements have several kinematic characteristics that depend on the direction of movement with respect to the direction of gravity (i.e., upward or downward movements). Upward movements tend to have a smaller proportion of acceleration time to deceleration time compared to the movements of equal distance and duration in the downward direction (Papaxanthis et al., 2005). On the other hand, peak and average speed of arm movements are not affected by the direction of movement (Papaxanthis et al., 2005).

Motor commands used for a specific movement in normogravity produce a different movement in other environments due to the different gravitational forces acting on the body. Motor commands planned for Earth’s environment produce lower movement responses in hypergravity and higher movement responses in microgravity (Bock et al., 1992). Moreover, arm movement studies in microgravity and hypergravity showed mixed results regarding the movement duration, pointing accuracy, and movement trajectory characteristics (Bock, 1998). Specifically, movement duration was shown to be longer in microgravity (Tafforin et al., 1989; Berger et al., 1997; Papaxanthis et al., 2005) and shorter in hypergravity (Bock et al., 1996) compared to normogravity. However, some experiments showed that movement duration of movements in hypergravity and microgravity did not differ from those in normogravity (Bringoux et al., 2012; Macaluso et al., 2017). In addition to the changes in movement duration, different gravitational conditions can also affect the accuracy of movements. There have been several studies that showed a decreased movement accuracy (Fisk et al., 1993) and pointing precision (Bock et al., 1992). It has been proposed that errors due to visual localization and proprioceptive information result in overshooting in hypergravity and undershooting in microgravity, while errors due to the inappropriate motor commands produce undershooting in hypergravity and overshooting in microgravity (Bock et al., 1992).

When movements are performed in a non-terrestrial environment, the CNS has to adapt to the new environment by taking into account the new gravitational force. This is especially relevant for astronauts and airplane pilots who encounter significant gravitoinertial variations. There are significant operational risks during the periods of altered gravitational environments, especially during the transitions between them (Shelhamer, 2016). To effectively operate a spacecraft or an airplane, it is important to have a proficient motor performance in all gravitational environments (Paloski et al., 2008). Altered sensorimotor functions affect fundamental skills required for operating the airplanes and spacecraft, such as timely reaching to switches on instrumental panels and smoothly guiding the trajectory of a vehicle (Paloski et al., 2008).

To mitigate the possible effects of changing gravitational levels, such as the changed movement duration and/or decreased accuracy, various methods of movement assistance could be applied. In their study, Weber et al. (2020) adapted haptic settings of a human-machine interface (joystick in this case) to mitigate changes caused by microgravity, however, this procedure did not produce satisfactory results. Moreover, Bringoux et al. (2012) showed that the effects of microgravity on arm movements can be mitigated by elastic bands that produce gravity-like torques in the shoulder joints. However, the results of this study are methodologically limited to the supine position of the body with an upward extended arm in which the gravity vector is aligned with the kinematical chain of the arm. It, therefore, remains largely unclear how a local compensation of gravitational force on the arm affects the movement characteristics, and whether such compensation could mitigate the effects of both stable and altered gravitational environment on motor control.

The main goals of our study are to investigate the effects of local gravity compensation on movement during altered environmental gravity and to decide whether assistive devices could be beneficial in these conditions. To address this, we designed a realistic pointing task experiment that participants performed while being subjected to the changing gravitational levels. Our experiment took place on an airplane during a set of parabolic flights which provide a suitable equivalent for a wide range of effects seen in orbital and deep-space flights (Shelhamer, 2016). The participants were seated and had to perform a series of pointing tasks on the screen in front of them while we systematically employed local gravity compensation at the arm with a motorized robotic device. To verify the efficiency of the local gravity compensation approach, we first identified the changes in movements caused by unfamiliar gravitational levels of microgravity and hypergravity with respect to the movements in normogravity and then investigated how these changes are affected by providing local gravity compensation at the arm.

## Materials and Methods

The study was performed during the 142nd CNES (French Space Agency) parabolic flight campaign that included 3 days of flights at Novespace-Merignac (France). Flights were composed of 31 parabolas, each consisting of three different gravitational conditions: normogravity (Earth gravity, ~1*g*), microgravity (~0*g*), and hypergravity (~1.8*g*).

### Participants

Nine right-handed participants (seven males and two females, mean ± SD; age 29.8 ± 7.4 years, height 176 ± 10.8 cm and body mass 71 ± 15.7 kg) took part in the study. None of them reported sensory or motor deficits. A medical examination qualified each participant for parabolic flights prior to participation. To avoid motion sickness, participants received medication (scopolamine) before boarding. It has been previously demonstrated that scopolamine utilization does not influence sensorimotor control of participants during parabolic flight (Ritzmann et al., 2016). None of the participants had previously experienced altered gravitational effects and they were all naive regarding the specific purpose of this experiment.

### Experimental Setup

**Figure 1:**
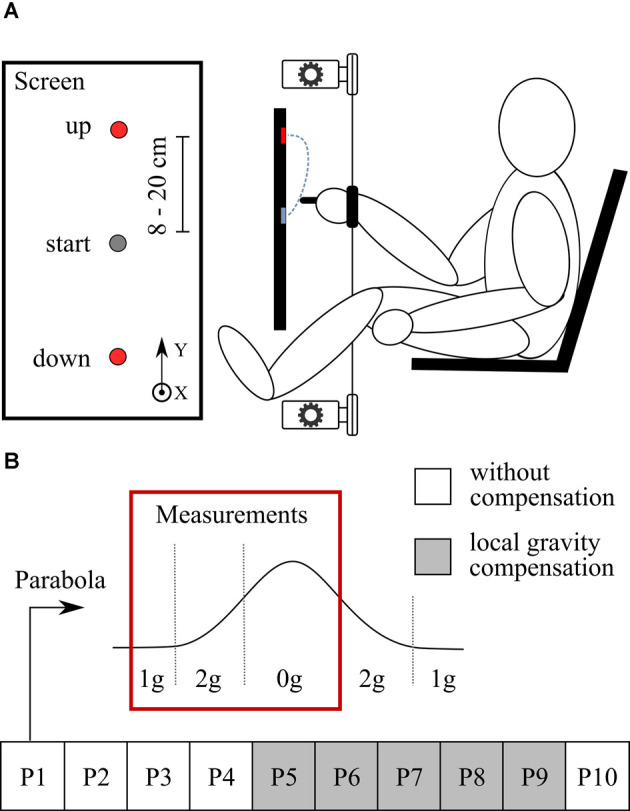
Experimental setup and protocol. **(A)** Participants performed pointing movements in a seated position. The goal was to hit a target presented on a screen in front of them. The participant’s arm was connected to the motors with thin strings. Motors were used to provide local gravity compensation at the arm. **(B)** Parabolic trajectory shows the flight path of the plane. The red box shows the part of the parabola when the measurements were taken. The experiment consisted of 10 parabolas; white squares show parabolas without local gravity compensation and gray squares show parabolas with local gravity compensation.

Participants were seated in front of a touchscreen display (display size 521 mm × 293 mm, ProLite T2435MSC-B2, Iiyama, Hoofddorp, Netherlands) oriented in a portrait mode as seen in Figure 1A. The seat was positioned low so that the participants’ legs were extended horizontally and their feet rested on the aircraft floor. To prevent displacement and floating of the body during the experiment, the body was tied to the backrest of the seat and the legs were tied to the floor with straps. Participants were using their right hand to hold a tactile stylus and were asked to perform a series of pointing movements from an initial position towards a target displayed either above or below the initial position. They were instructed to perform the movements as accurately and as fast as possible. The initial position was displayed on the screen as a gray circle 60 mm in diameter and was located approximately at shoulder height. The target was displayed on the screen as a red circle 20 mm in diameter. There were seven upper and seven lower targets, positioned either 8, 10, 12, 14, 16, 18, or 20 cm from the initial position in either direction. They were aligned on a vertical axis in front of the participant’s right shoulder. Only one target was displayed for a given pointing movement. To avoid possible anticipation of the target location, the targets were displayed in a pseudo-random order where each target was represented an equal number of times, but the sequence was long enough to prevent memorization and therefore any anticipation effects. The motion of the stylus was measured by recording the position of a reflective marker placed on the stylus using a contactless motion capture system (Vicon, Yarnton, UK) recording at 100 Hz sampling frequency. Moreover, the exact location of where the stylus touched the display was acquired by the touchscreen interface. Additionally, we measured the muscular activity of deltoid anterior, deltoid posterior, trapezius, and pectoralis of the right arm using an EMG system (SX230 sensor, Biometrics Ltd, New- port, UK). Raw signals were acquired with a sampling frequency of 1,000 Hz.

In some conditions (defined in experimental protocol) a motorized robotic device was used to provide local gravity compensation at the arm. Two motors (EMMS-AS-55-S-TM, Festo, Esslingen, Germany), positioned above and below the participant’s arm, were connected to two thin strings (Dyneema^®^ 1.5 mm, YSM and Partners, Dobra, Poland) that ensured a negligible extension with respect to the movement amplitude. The strings were further attached to a large Velcro strap that was strapped around the participant’s right wrist. When gravity compensation was provided, the motors generated limited vertical forces (the force was limited to 30 N in either direction in order to ensure safety while allowing for full support of the arm) in order to locally re-establish normogravity environment at the wrist, and hence normal gravitational torque at the shoulder joint. The motors generated forces to lighten or add weight on the wrist in hypergravity or microgravity conditions, respectively. The force required to keep the arm in a horizontal position was measured beforehand for each participant (18.6 ± 4.8 N) and was used to compensate for the weight of the arm so that the torque felt by the participant in the shoulder joint was equal to that felt in normogravity. To control the motors in closed-loop according to the experimental conditions, a three-dimensional accelerometer (Xsens, Enschede, Netherlands), fixed on the floor of the aircraft, recorded the ambient gravitational phase and transferred the signal to the motor controllers in real-time with a rate of 1 kHz. When gravity compensation was not provided, a constant pretension force of 10 N was applied by both motors in the opposite direction to prevent the string slack.

### Experimental Protocol

Participants completed 10 successive parabolas, during which they were exposed to the normogravity, hypergravity, and microgravity environments, also referred to as 1*g*, 2*g*, and 0*g*, respectively. The data recording period in each parabola consisted of a steady flight phase 20 s before the entry in the parabola (normogravity), the pull-up phase (hypergravity), and the weightlessness phase (microgravity) as shown in Figure 1B. During this period, participants performed continuous pointing movements that lasted for around 1 min. They rested during the remaining time of the parabola and in between parabolas (ca 1 min). During the first four parabolas (P1 to P4) and the last parabola (P10), participants experienced all gravitational conditions without any compensation. From P5 to P9, local gravity compensation was enabled. When compensation was used, participants experienced constant local normogravity at the wrist while the body was immersed into the changing environmental gravity conditions.

### Data Processing and Statistical Analysis

Arm movements were analyzed by looking at the movement duration, accuracy, shape of the trajectory, movement symmetry, and muscle activity for each pointing movement. We defined movement onset as the time when the stylus left the initial position on the screen and the end when the stylus touched the screen again. The start and the end of the movement were calculated based on the data from the touch screen since that gave us the most accurate timeframe of the movement. We calculated movement duration as the time between movement onset and offset. To analyze the accuracy of pointing, we looked at the location of the hit with respect to the target location calculated as a vertical distance between the center of the target and the position where the stylus touched the screen. We looked at the absolute deviations as well as signed deviations of the hits, where a positive deviation represents a hit above the target and a negative deviation represents a hit under the target. If the hit was above the target for upward movements or under the target for downward movements, the target was overshot. Contrary, if the hit was under the target for upward movements or above the target for downward movements, the target was undershot. Movements with an absolute deviation greater than 20 mm (which is the distance between the two targets) were excluded from the analysis. Moreover, to analyze the kinematics of arm movements, we looked at the shape of the trajectory and movement symmetry. The shape of the trajectory was assessed by determining trajectory curvature calculated as a maximal deviation of the trajectory in the horizontal direction, while movement symmetry was assessed by determining the relative time to peak velocity (rTPV) obtained by dividing time to peak velocity by movement duration. Both the trajectory curvature and rTPV were calculated based on marker position data. Marker positions were interpolated for missing data and low pass filtered with 2nd order Butterworth filter (zero phase lag, 10 Hz cut-off frequency). We excluded data where environmental or hand simulated gravity level changed during a single trial, the standard deviation of stationary markers on the screen exceeded 4 mm, there were more than five consecutive instances of missing markers in the raw data, there were discontinues jumps in marker data, the movement did not start in the area of start target, stylus marker moved in the opposite direction of the target, or stylus marker trajectory was abnormal. Finally, muscle activity was analyzed by calculating integrated EMG (iEMG). EMG data was band-pass filtered with 2nd order Butterworth filter (zero phase lag) with 20 Hz and 350 Hz cut-off frequencies. Afterward, the EMG envelope was calculated and the signal was integrated over time for each movement to determine muscular effort.

To compare the measured parameters across different conditions we conducted a linear mixed models analysis with three gravity conditions (1*g*, 0*g*, 2*g*) × 2 ompensation conditions (local gravity compensation, no compensation) × 7 targets statistical design. For EMG analysis we used linear mixed models analysis with 3 gravity conditions (1*g*, 0*g*, 2*g*) × 2 compensation conditions (local gravity compensation, no compensation) × 4 muscles (deltoid anterior, deltoid posterior, trapezius, and pectoralis) statistical design. The statistical analysis was conducted in R (R Core Team, 2020) with the nlme (Pinheiro et al., 2020) and multcomp (Hothorn et al., 2008) packages, while all other analyses were conducted in Matlab (Mathworks, Natick, MA, USA). The focus of the experiment was to study the effects of changed gravity on human arm movements and how local gravity compensation mitigates these effects, therefore we were mainly interested in the main effect of gravity and the interaction effect between gravity and compensation. Additionally, the analysis was performed separately for both directions and we did not directly compare downward and upward movements. *Post hoc* *t*-tests with Bonferroni correction were conducted to determine the significant differences between the specific conditions. To determine the effects of gravity, we compared microgravity and hypergravity to normogravity (1*g*–0*g* and 1*g*–2*g*). Moreover, to determine if changes caused by the changed gravity can be decreased with the local gravity compensation, we compared the conditions without compensation to the conditions with local gravity compensation (0*g*–0*gC* and 2*g*–2*gC*). We further compared the conditions with local gravity compensation in microgravity and hypergravity to normogravity to see if the effects of the gravitational change were eliminated (1*g*–0*gC* and 1*g*–2*gC*). The level of statistical significance was set at 0.05. For statistical analysis, we used averaged values of each target for all parameters. However, for visual presentation, we calculated average values across targets and only present values of each target for accuracy and shape of the trajectory.

## Results

Our experiment investigated the effects of local gravity compensation on movements during microgravity and hypergravity. It took place on an airplane during a set of parabolic flights. The participants were holding a tactile stylus and performed a series of pointing movements towards the screen in front of them from an initial position towards a target displayed either above or below the initial position. We systematically employed local gravity compensation at the arm with a motorized robotic device. To investigate the effects of local gravity compensation on the pointing performance, we looked at the duration and accuracy of movements. Moreover, to determine the effects of gravity compensation on the kinematics of arm movement, we analyzed the shape of arm movement and the corresponding velocity profiles. Finally, muscle activity was investigated to determine whether muscle activation patterns during local gravity compensation resemble those in normogravity.

### Movement Duration

**Figure 2:**
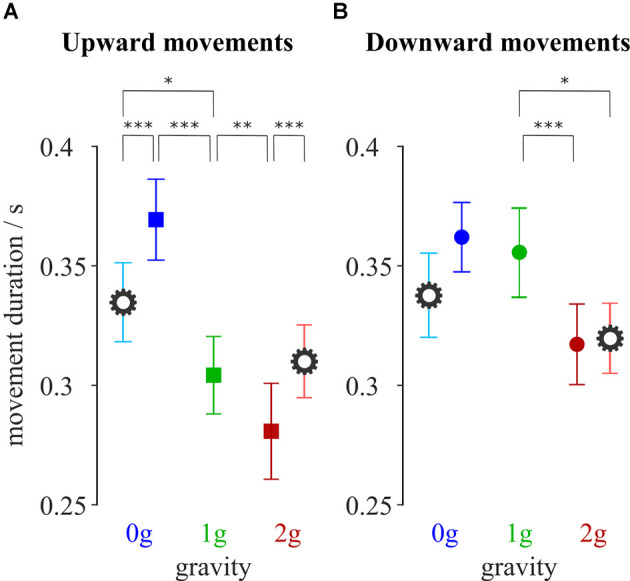
Average movement duration for upward **(A)** and downward **(B)** movements. Blue-shaded colors represent movements in microgravity, green-shaded colors represent movements in normogravity while red-shaded colors represent movements in hypergravity. Conditions with local gravity compensation are shown in light colors and are emphasized with a gear sign. Error bars show the standard error of mean. *p < 0.05, **p < 0.01, ***p < 0.001.

Participants performed upward and downward movements towards the targets of different distances. Movement duration for each combination of gravity and compensation conditions averaged for all targets, is shown in Figure 2.

The gravitational changes had a significant effect on the duration of movement. This was confirmed by the analysis of variance that showed a main effect of gravity on the movement duration for both upward (*F*_(2,312)_ = 61.72, *p* < 0.001) and downward (*F*_(2,302)_ = 16.78, *p* < 0.001) movements. Additionally, there was a significant interaction between gravity and compensation for both the upward (*F*_(2,312)_ = 18.32, *p* < 0.001) and downward (*F*_(2,302)_ = 4.79, *p* = 0.009) movements.

*Post hoc* analyses showed that upward movements in microgravity took a longer time compared to movements in normogravity (Table 1). On the other hand, movements in hypergravity took less time compared to movements in normogravity which was statistically significant for both upward and downward movements.

**Table 1:** *Post hoc* analysis of movement duration.

	MICROGRAVITY			HYPERGRAVITY		
	Comparison	*z* value	*p* value	Comparison	*z* value	*p* value
UP	1*g*–0*g*	−8.322	**<0.001**	1*g*–2*g*	3.240	**0.008**
	0*g*–0*gC*	5.114	**<0.001**	2*g*–2*gC*	−3.494	**0.003**
	1*g*–0*gC*	−3.102	**0.013**	1*g*–2*gC*	−0.287	1.000
DOWN	1*g*–0*g*	−0.535	1.000	1*g*–2*g*	4.956	**<0.001**
	0*g*–0*gC*	2.573	0.071	2*g*–2*gC*	−1.604	0.760
	1*g*–0*gC*	2.078	0.264	1*g*–2*gC*	3.028	**0.017**

*Results of *post hoc* tests (*z* value and *p* value) for every specified pair of conditions. *p* values that are statistically significant are shown in bold*.

Local gravity compensation decreased movement duration in microgravity and increased it in hypergravity, however, this was only statistically significant for the upward movements (Table 1).

The impact of local gravity compensation resulted in values for movement duration during microgravity and hypergravity that were closer to the values observed in normogravity. *Post hoc* analysis showed no statistical differences between hypergravity with local gravity compensation and normogravity for upward movements, as well as no statistical difference between microgravity with local gravity compensation and normogravity for downward movements. Additionally, the movement duration of movements with local gravity compensation in microgravity was reduced compared to movements without local gravity compensation, yet still remained longer than the movement duration for movements in normogravity.

### Accuracy

**Figure 3:**
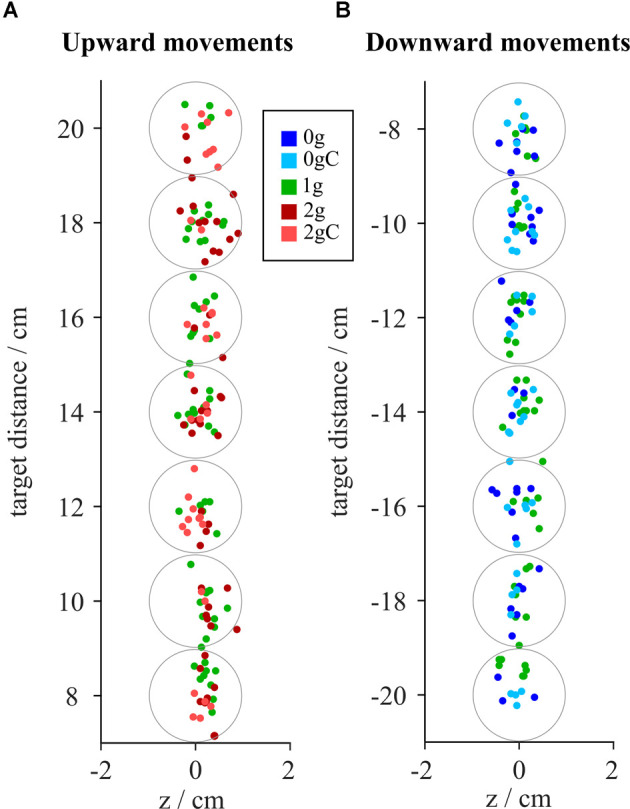
Distributionof hits around the target points from a representative subject for upward **(A)** and downward **(B)** movements. Gray circles represent the target area. Blue-shaded colors represent movements in microgravity, green-shaded colors represent movements in normogravity while red-shaded colors represent movements in hypergravity. Conditions with local gravity compensation are shown in light colors.

**Figure 4:**
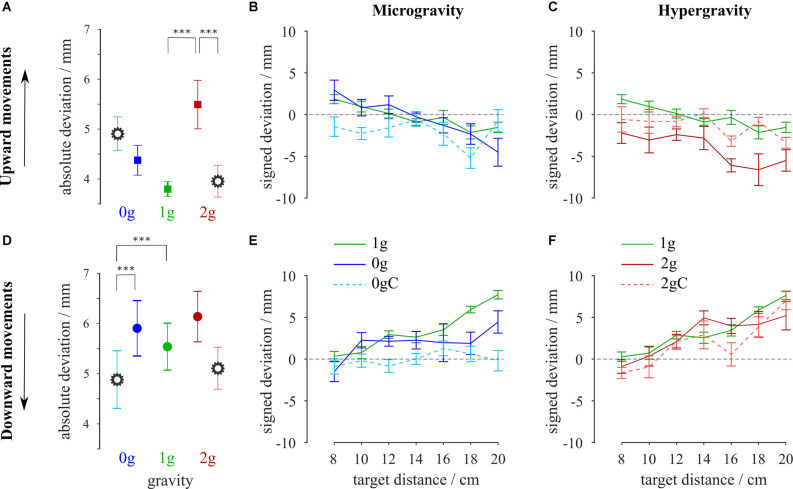
Average absolute deviations for upward **(A)** and downward **(D)** movements. Signed deviations for every target in microgravity **(B,E)** and hypergravity **(C,F)** for upward and downward movements, respectively. Blue-shaded colors represent movements in microgravity, green-shaded colors represent movements in normogravity while red-shaded colors represent movements in hypergravity. Conditions with local gravity compensation are shown in light colors and are emphasized with a gear sign. Error bars show the standard error of mean. ***p < 0.001.

To investigate the accuracy of pointing, we looked at the location of the hits on the screen on which the targets were displayed (Figure 3). The absolute deviations of the hits with respect to the target location for each combination of gravity and compensation conditions, averaged for all targets, are shown in Figures 4A,D. Moreover, the signed deviations of the hits for the individual targets are shown separately in Figures 4B,E for microgravity, and in Figures 4C,F for hypergravity. All statistical analyses for accuracy were performed on signed deviation values.

The analysis of variance revealed a main effect of gravity on accuracy for both upward (*F*_(2,312)_ = 61.72, *p* < 0.001) and downward (*F*_(2,302)_ = 19.96, *p* < 0.001) movements. Moreover, there was a significant interaction between gravity and compensation on signed deviation of the hits for the upward (*F*_(2,312)_ = 18.60, *p* < 0.001) and downward (*F*_(2,302)_ = 5.29, *p* = 0.005) movements.

Accuracy of movements in microgravity and hypergravity slightly decreased compared to movements in normogravity, which is shown as the increase in absolute deviations. Notably, *post hoc* analysis showed a significant difference in accuracy between the normogravity and hypergravity conditions for the upward movements (Table 2). Upward movements in hypergravity had a negative signed deviation that clearly shows an undershoot in pointing movements (Figure 4C). However, there was no statistical difference in the accuracy of downward movements between hypergravity and normogravity, as well as in the accuracy of both the upward and downward movements between microgravity and normogravity.

**Table 2:** *Post hoc* analysis of signed deviations of the hits.

	MICROGRAVITY			HYPERGRAVITY		
	Comparison	*z* value	*p* value	Comparison	*z* value	*p* value
UP	1*g*–0*g*	−0.234	1.000	1*g*–2*g*	5.729	**<0.001**
	0*g*–0*gC*	2.007	0.313	2*g*–2*gC*	−4.586	**<0.001**
	1*g*–0*gC*	1.783	0.522	1*g*–2*gC*	1.114	1.000
DOWN	1*g*–0*g*	−2.060	0.276	1*g*–2*g*	−0.467	1.000
	0*g*–0*gC*	−3.890	**<0.001**	2*g*–2*gC*	−0.945	1.000
	1*g*–0*gC*	−6.025	**<0.001**	1*g*–2*gC*	−1.396	1.000

*Results of post hoc tests (*z* value and *p* value) for every specified pair of conditions. *p* values that are statistically significant are shown in bold*.

*Post hoc* analysis showed an increase in the signed deviation for upward movements in hypergravity with local gravity compensation compared to movements in hypergravity without compensation (Table 2). This resulted in eliminating the undershoot observed in upward movements in hypergravity without compensation. Comparison between movements in hypergravity with local gravity compensation and normogravity revealed no statistical differences for both directions of movement. Additionally, there was a decrease in the signed deviation for the downward movements in microgravity with local gravity compensation compared to movements in microgravity without compensation. The location of the hits of movements in microgravity with local gravity compensation was closer to the center of the target. Comparison between movements in microgravity with local gravity compensation and normogravity showed a statistical difference for downward movements and no statistical difference for upward movements (Table 2).

### Movement Trajectory

**Figure 5:**
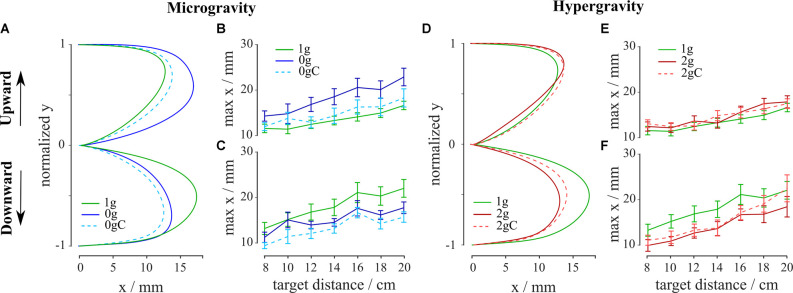
Average trajectories, normalized in the vertical direction, for upward **(A)** and downward **(D)** movements. Maximal deviation of trajectories (max x) for every target in microgravity **(B,C)** and hypergravity **(E,F)** for upward and downward movements, respectively. Blue-shaded colors represent movements in microgravity, green-shaded colors represent movements in normogravity while red-shaded colors represent movements in hypergravity. Conditions with local gravity compensation are shown in light colors and with dotted lines. Error bars show the standard error of mean.

To investigate the effects of environmental gravity, the direction of movement, and local gravity compensation on the shape of the arm movements, we looked at the trajectory curvature. By comparing the arm movement trajectories in microgravity with those in normogravity irrespective of local gravity compensation, we observed larger curvatures towards the trunk for the upward movements and lower curvatures for the downward movements (green vs. blue shaded lines in Figures 5A–C). On the other hand, by comparing the shapes of trajectories in hypergravity with those in normogravity, we observed similar curvatures for the upward movements and smaller curvatures for the downward movements **(**green vs. red shaded lines in Figures 5D–F).

The analysis of variance showed a main effect of gravity on the curvature for both upward (*F*_(2,312)_ = 41.27, *p* < 0.001) and downward (*F*_(2,302)_ = 86.21, *p* < 0.001) movements. Moreover, we investigated the effects of local gravity compensation on the movement trajectories in both microgravity and hypergravity. The analysis of variance revealed a significant interaction between gravity and compensation on trajectory curvature for both upward (*F*_(2,312)_ = 12.91, *p* < 0.001) and downward (*F*_(2,302)_ = 9.36, *p* = 0.001) movements.

Looking at the movements without the local gravity compensation, *post hoc* analysis showed that there was a significant difference between normogravity and microgravity conditions for both movement directions and between normogravity and hypergravity conditions for the downward movements (Table 3).

**Table 3:** *Post hoc* analysis of trajectory curvatures.

	MICROGRAVITY			HYPERGRAVITY		
	Comparison	*z* value	*p* value	Comparison	*z* value	*p* value
UP	1*g*–0*g*	−9.081	**<0.001**	1*g*–2*g*	−1.450	1.000
	0*g*–0*gC*	7.035	**<0.001**	2*g*–2*gC*	0.208	1.000
	1*g*–0*gC*	−1.925	0.380	1*g*–2*gC*	−1.251	1.000
DOWN	1*g*–0*g*	7.369	**<0.001**	1*g*–2*g*	8.898	**<0.001**
	0*g*–0*gC*	3.526	**0.003**	2*g*–2*gC*	−2.522	0.081
	1*g*–0*gC*	10.986	**<0.001**	1*g*–2*gC*	5.798	**<0.001**

*Results of *post hoc* tests (*z* value and *p* value) for every specified pair of conditions. *p* values that are statistically significant are shown in bold*.

Local gravity compensation significantly decreased the curvature of the movements in microgravity in both directions (blue shaded lines in Figures 5A–C), but on the other hand, the curvature of movements in hypergravity remained largely unchanged (red shaded lines in Figures 5D–F). Comparison of trajectory curvatures during local gravity compensation with those in normogravity revealed no statistical differences between normogravity and microgravity and between normogravity and hypergravity conditions for the upward movements and significant differences between the same conditions for the downward movements (Table 3).

**Figure 6:**
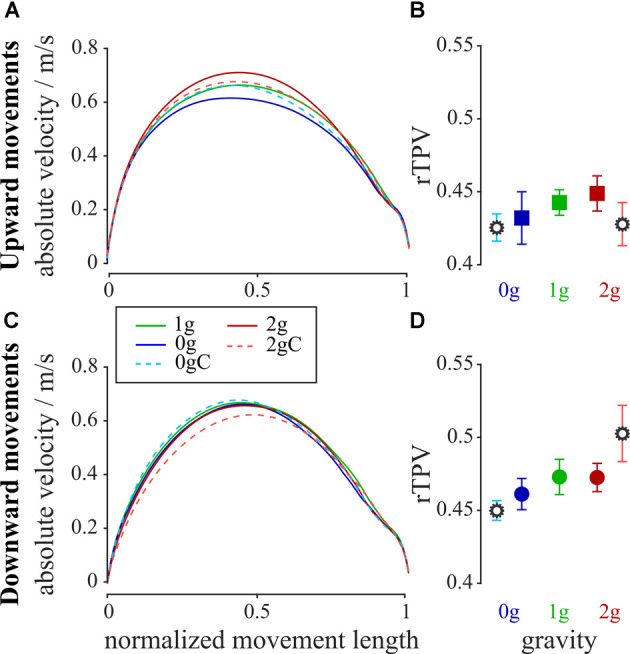
Average absolute velocity profiles for the upward **(A)** and downward **(C)** movements together with the average rTPV for all gravity conditions for the upward **(B)** and downward **(D)** movements. Blue-shaded colors represent movements in microgravity, green-shaded colors represent movements in normogravity while red-shaded colors represent movements in hypergravity. Conditions with local gravity compensation are shown in light colors and are emphasized with a gear sign. Error bars show the standard error of mean.

To get a further insight into the changes of the arm movements, we looked at the rTPV which characterizes the symmetry of the trajectories. Figure 6 shows the velocity profiles and rTPV for all combinations of gravity and compensation conditions and for both movement directions. Mean rTPV in normogravity for the upward movements was 0.44 ± 0.01 and 0.47 ± 0.01 for the downward movements. The analysis of variance showed no main effect of gravity (*F*_(2,312)_ = 1.52, *p* = 0.220) and no interaction between gravity and compensation (*F*_(2,312)_ = 1.63, *p* = 0.197) on rTPV of the upward movements. However, there was a main effect of gravity (*F*_(2, 302)_ = 8.52, *p* < 0.001) and an interaction between gravity and compensation (*F*_(2,302)_ = 3.66, *p* = 0.027) on rTPV of the downward movements, yet the *post hoc* tests showed no statistically significant differences for the relevant comparisons.

### Muscle Activity

**Figure 7:**
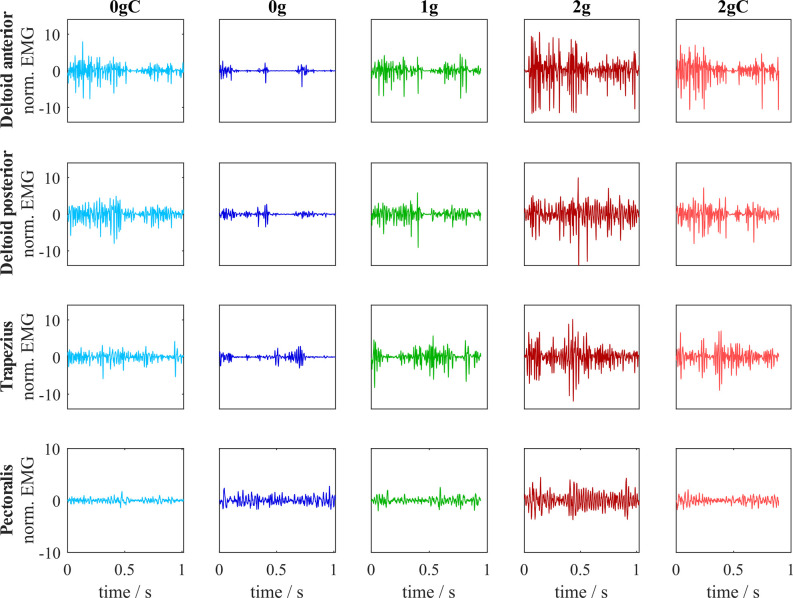
Normalized EMG signals for all muscles across different gravitational and compensation conditions. This data corresponds to one representative subject for the target furthest away in the upward direction.

**Figure 8:**
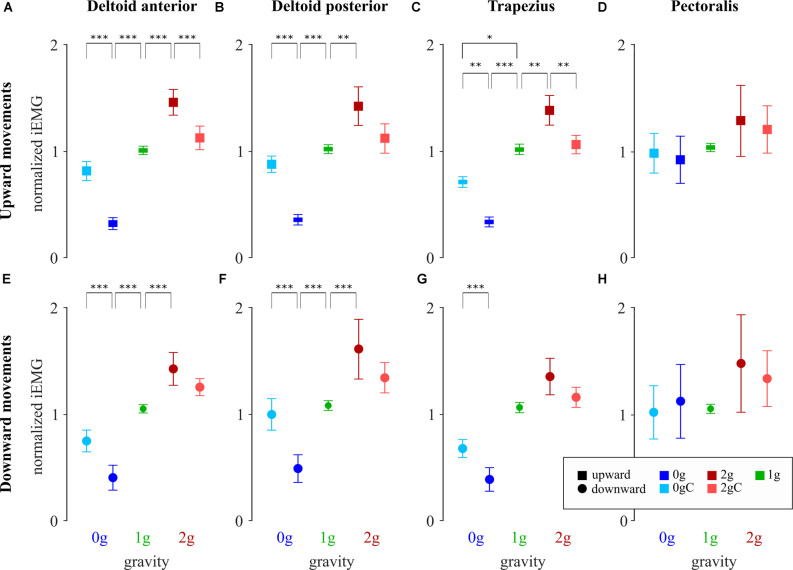
Normalized iEMG of deltoid anterior **(A,E)**, deltoid posterior **(B,F)**, trapezius **(C,G)**, and pectoralis **(D,H)** for upward and downward movements, respectively. Blue-shaded colors represent movements in microgravity, green-shaded colors represent movements in normogravity while red-shaded colors represent movements in hypergravity. Conditions with local gravity compensation are shown in light colors. Squares denote upward movements and circles denote downward movements. Error bars show the standard error of mean. *p < 0.05, **p < 0.01, ***p < 0.001.

We recorded EMG of the major shoulder muscles: deltoid anterior, deltoid posterior, trapezius, and pectoralis (Figure 7). To determine whether environmental gravity, the direction of movement, and local gravity compensation have an effect on the muscular effort, we calculated the iEMG for each arm movement (Figure 8).

The analysis of variance revealed a significant main effect of gravity on iEMG of the upward movements for deltoid anterior (*F*_(2,33)_ = 75.81, *p* < 0.001), deltoid posterior (*F*_(2,33)_ = 27.67, *p* = 0.001) and trapezius (*F*_(2,33)_ = 45.55, *p* < 0.001), but not for pectoralis (*F*_(2,32)_ = 3.08, *p* = 0.06). There was a significant main effect of gravity on iEMG of the downward movements for deltoid anterior (*F*_(2,32)_ = 41.74, *p* < 0.001), deltoid posterior (*F*_(2,32)_ = 16.27, *p* < 0.001) and trapezius (*F*_(2,32)_ = 26.23, *p* < 0.001), but again not for pectoralis (*F*_(2,31)_ = 2.43, *p* = 0.10). With the exception of pectoralis, iEMG decreased in microgravity and increased in hypergravity compared to normogravity for movements in both directions. Specific comparisons are given in Table 4. The analysis of variance further revealed interaction between gravity and compensation on iEMG of the upward movements for deltoid anterior (*F*_(2,33)_ = 28.23, *p* < 0.001), deltoid posterior (*F*_(2,33)_ = 14.16, *p* = 0.001), and trapezius (*F*_(2,33)_ = 14.91, *p* < 0.001), but not for pectoralis (*F*_(2,32)_ = 0.20, *p* = 0.82). Similarly, there was an interaction between gravity and compensation for iEMG of the downward movements for deltoid anterior (*F*_(2,32)_ = 10.02, *p* < 0.001), deltoid posterior (*F*_(2,32)_ = 8.19, *p* = 0.001) and trapezius (*F*_(2,32)_ = 5.96, *p* = 0.006), but not for pectoralis (*F*_(2,31)_ = 0.41, *p* = 0.67).

**Table 4:** *Post hoc* analysis of iEMG.

	MICROGRAVITY			HYPERGRAVITY		
	Comparison	*z* value	*p* value	Comparison	*z* value	*p* value
**DELTOID ANTERIOR**
UP	1*g*–0*g*	8.466	**<0.001**	1*g*–2*g*	−5.922	**<0.001**
	0*g*–0*gC*	−5.542	**<0.001**	2*g*–2*gC*	4.475	**<0.001**
	1*g*–0*gC*	2.609	0.063	1*g*–2*gC*	−1.434	1.000
DOWN	1*g*–0*g*	5.847	**<0.001**	1*g*–2*g*	−3.587	**<0.001**
	0*g*–0*gC*	−3.292	**<0.001**	2*g*–2*gC*	1.996	0.322
	1*g*–0*gC*	2.301	0.150	1*g*–2*gC*	−1.255	1.000
**DELTOID POSTERIOR**
UP	1*g*–0*g*	5.442	**<0.001**	1*g*–2*g*	−3.551	**0.003**
	0*g*–0*gC*	−4.051	**<0.001**	2*g*–2*gC*	2.628	0.060
	1*g*–0*gC*	1.155	1.000	1*g*–2*gC*	−0.912	1.000
DOWN	1*g*–0*g*	3.341	**<0.001**	1*g*–2*g*	−3.083	**<0.001**
	0*g*–0*gC*	−3.079	**<0.001**	2*g*–2*gC*	1.733	0.582
	1*g*–0*gC*	0.082	1.000	1*g*–2*gC*	−1.059	1.000
**TRAPEZIUS**
UP	1*g*–0*g*	6.952	**<0.001**	1*g*–2*g*	−3.759	**0.001**
	0*g*–0*gC*	−3.598	**0.002**	2*g*–2*gC*	3.251	**0.008**
	1*g*–0*gC*	3.138	**0.012**	1*g*–2*gC*	−0.455	1.000
DOWN	1*g*–0*g*	5.059	**<0.001**	1*g*–2*g*	−2.236	0.177
	0*g*–0*gC*	−2.143	0.225	2*g*–2*gC*	1.520	0.900
	1*g*–0*gC*	2.673	0.053	1*g*–2*gC*	−0.498	1.000

*Results of *post hoc* tests (*z* value and *p* value) for every specified pair of conditions. *p* values that are statistically significant are shown in bold*.

Comparing the iEMG during local gravity compensation in microgravity and hypergravity with iEMG during normogravity (Table 4) indicates that, with an exception of trapezius during the upward motion, local gravity compensation largely mitigates the effects of altered environmental gravity on the muscular effort of the major shoulder muscles.

## Discussion

The main purpose of this study was to investigate if local gravity compensation could mitigate the well-known changes to arm movements caused by novel gravitational environments (Tafforin et al., 1989; Bock et al., 1992, 1996; Fisk et al., 1993; Berger et al., 1997; Papaxanthis et al., 2005; Ritzmann et al., 2019). In fact, the altered environmental gravity during our experiment significantly affected most of the observed parameters of arm movements with respect to normogravity. Our method of local gravity compensation was able to mitigate some of these changes in the observed parameters caused by the novel gravitational environment.

### Changes Due to Novel Environmental Gravity

We first identified the changes in movements caused by unfamiliar environmental gravity. With respect to the movements in normogravity, most of the observed parameters, e.g., the movement duration, accuracy, shape of the trajectory, and muscle activity were significantly changed. The only parameter that was not affected by the altered environmental gravity was movement symmetry.

We observed changes in movement duration that are consistent with previous studies that showed increased movement duration in microgravity (Tafforin et al., 1989; Berger et al., 1997; Papaxanthis et al., 2005) and decreased movement duration in hypergravity (Bock et al., 1996) compared to normogravity. During microgravity, participants did not feel the weight of the tactile stylus as well as the weight of their arm. The sensorimotor system could have misunderstood the absence of weight as the absence of the stylus’s mass which resulted in reduced motor commands. The unchanged mass and reduced acceleration consequentially lead to a longer movement duration. During hypergravity, participants felt an increased weight of the tactile stylus and their arm. Increased motor commands, therefore, lead to shorter movement duration.

The accuracy of pointing movements in microgravity was not affected, while accuracy in hypergravity worsened. There was a decrease of pointing accuracy in hypergravity compared to the accuracy in normogravity, however, it is significantly different only for the upward movements. To a certain degree, this is consistent with the studies that showed decreased accuracy (Fisk et al., 1993) and pointing precision (Bock et al., 1992) of movements in hypergravity. There was an undershoot observed during the upward movements in hypergravity. Participants tended to point lower with respect to the center of the target. This might be due to the under compensation of the extra weight that the participants experienced at the hand or due to the lower responses in hypergravity. In contrast to hypergravity, microgravity did not affect the accuracy. The duration of movements was longer compared to the duration of movements in normogravity. Consequently, the prolonged feedback likely helped with accuracy.

Changes in the environmental gravity had a significant effect on the shape of the movement trajectory. Upward movements in microgravity had a larger curvature while downward movements had a smaller curvature towards the trunk compared to movements in normogravity. Similar changes were previously observed in upward and downward arm movements, where movement trajectory in microgravity shifted away from the trunk for upward movements and closer to the trunk for downward movements compared to movements in normogravity (Papaxanthis et al., 1998).

On the other hand, upward movements in hypergravity had a comparable curvature to upward movements in normogravity while downward movements had a smaller curvature than downward movements in normogravity and, interestingly, similar to those in microgravity.

Muscle activations were lower during the arm movements in microgravity and higher in hypergravity with respect to the corresponding muscle activations in normogravity. This is in line with a previous study where they showed that increased environmental gravity increases EMG amplitudes (Ritzmann et al., 2019). Changes in muscle activations were observed in deltoid anterior, deltoid posterior, and trapezius for movements in both directions. During microgravity, the weight of the participant’s arm and stylus was reduced therefore the muscle activity needed for the movement was lower. On the contrary, the weight of the arm and stylus in hypergravity was increased and the muscle activity needed for a successful movement was also increased. However, there were no changes in the muscle activity of the pectoralis, probably because it acts primarily perpendicular to the gravitational vector.

### Effects of Local Gravity Compensation

We further verified the effects of the local gravity compensation approach on arm movement characteristics, and whether such compensation could mitigate the effects of altered environmental gravity on motor control. Additionally, we wanted to check whether movement symmetry, which was not affected by altered environmental gravity would be affected by local gravity compensation. The use of local gravity compensation significantly affected arm movements with respect to the same gravity conditions without compensation. Specifically, it affected movement durations of upward movement, improved overall accuracy of movements, and restored muscle activations to values observed in normogravity. On the other hand, movement symmetry, which was not affected by changed environmental gravity was not affected by local gravity compensation and remained the same as in normogravity.

With respect to uncompensated movements, movements in microgravity with local gravity compensation took less time, while movements in hypergravity took a longer time. When compared to movements in normogravity, movements with local gravity compensation had similar movement duration for both microgravity and hypergravity conditions. This shows the effectiveness of local gravity compensation in regards to restoring movement duration observed in normogravity. We presume that, by applying the external force with the motors, the proprioceptive feedback was augmented and participants were better able to estimate the mass of the stylus and their arm. Motor commands were, therefore, neither reduced nor increased, which resulted in similar movement duration as for the normogravity condition. This shows that proprioceptive feedback contributes indispensable information for generating suitable motor commands and should therefore not be underestimated.

Movements in microgravity with local gravity compensation had similar accuracy while movements in hypergravity were more accurate compared to uncompensated movements. When compared to movements in normogravity, the accuracy of movements with local gravity compensation was similar or even improved. Local gravity compensation improved the accuracy in hypergravity, especially for the upward movement. Gravity compensation compensated for the extra weight and the movements likely became easier to perform. On the other hand, there was no statistical difference in signed deviations between movements in normogravity and microgravity, however local gravity compensation still had an effect on the accuracy of movements in microgravity. Movements in microgravity with local gravity compensation had smaller signed deviations compared to movements in microgravity without compensation as well as compared to normogravity, meaning that in microgravity participants tended to point closer to the target when local gravity compensation was provided. Moreover, differences between targets mostly disappeared which could be an additional benefit of gravity compensation that we used. Local gravity compensation in microgravity added a downward force which might be a reason why hit dispersions shifted downwards and closer to the center of the target. Another possibility is that participants were able to easily dissociate the effects of local gravity compensation and its effects on the movement control and take advantage of it.

Both upward and downward movements in microgravity with local gravity compensation had a smaller curvature compared to movements in microgravity without compensation. On the other hand, movements in hypergravity with local gravity compensation had a similar curvature as movements in hypergravity without compensation. When compared to movements in normogravity, upward movements with local gravity compensation in microgravity as well as in hypergravity had similar curvatures. Downward movements with local gravity compensation in both microgravity and hypergravity had smaller curvature compared to movements in normogravity. Local gravity compensation affected the shape of the trajectory in microgravity but not in hypergravity. In microgravity, local gravity compensation reduced the curvatures of both upward and downward movements. We can conclude that local gravity compensation had an effect on the movement trajectories but did not mitigate the changes due to the altered environmental gravity.

The only parameter that was not affected by altered environmental gravity was movement symmetry. Velocity profiles as well as rTPV, for both conditions with local gravity compensation compared to normogravity condition, showed no undesired effects of using gravity compensation during unfamiliar environmental gravity.

Lastly, muscle activations in microgravity and hypergravity were significantly affected by the decreased or increased gravitational forces exerted on the limb. Our method of compensation restored normal gravitational constraints at the shoulder joint by adding or subtracting the appropriate amount of torque. The beneficial use of gravity compensation was previously observed in rehabilitation systems with arm-weight support where they reduced muscle activity and preserved muscle synergies (Prange et al., 2009; Coscia et al., 2014). Our results show how local gravity compensation could be used to restore normal gravity muscle activations while preserving muscle synergies in novel gravitational environments.

### Conclusion

It has been previously hypothesized and showed that the central nervous system contains an internal representation of gravitational torques used for sensorimotor predictions (Gentili et al., 2009). Additionally, due to our evolutionary process, motor commands are optimized with respect to the effect of gravity on our body (Berret et al., 2008; Crevecoeur et al., 2009; Gaveau and Papaxanthis, 2011). By restoring the shoulder torque and consequently muscle activations to normogravity levels, we provided the CNS with additional proprioceptive information, and reestablished a more familiar environment, in order to prepare an appropriate motor plan for executing the movements necessary to complete the task. This resulted in more comparable movement durations with respect to the normogravity as well as improved accuracy of performing the task. Our results further confirmed the findings from Bringoux et al. (2012), where they showed normal gravity arm torque contributes to appropriate motor planning.

The improvement however was not complete, since there was still some sensory conflict between the proprioceptive feedback from the arm and the information gathered from the vestibular system. Moreover, the gravitational compensation was induced by vertical forces that only acted on a single part of the arm and were not distributed over the whole upper limb. This could be the reason why gravity compensation did not mitigate the changes to the shape of the trajectory and why some observed parameters, despite the improvement, did not reach the same values as in normogravity. Nevertheless, the proprioceptive feedback appears to have high importance in generating appropriate motor planning, since we saw significant changes in the movement parameters with local gravity compensation during which, the vestibular system was still experiencing microgravity or hypergravity effects.

We showed how a local gravity compensation system could be effectively used to mitigate undesired effects while performing motion in altered gravitational levels. We showed that local gravity compensation significantly alleviates the deviations of movement duration and muscle activations due to the altered environmental gravity and improves the accuracy of pointing. Overall, the results of our study strongly suggest that local compensation systems have a high potential to assist humans during movements in environments where gravity is different from what we daily experience on Earth.

## Data Availability Statement

The raw data supporting the conclusions of this article will be made available by the authors, without undue reservation.

## Ethics Statement

The studies involving human participants were reviewed and approved by Committee for Personal Protection (CPP). The patients/participants provided their written informed consent to participate in this study.

## Author Contributions

GB, DN, CP, OW, and JB designed the study. MJ, MB, JB, OW, and GB performed the experiment. TK, MJ, and MB analyzed the data. TK and MJ wrote the manuscript. MJ, OW, JB, GB, DN, and CP gave feedback on the manuscript. MJ, TK, GB, DN, CP, OW, and JB read and approved the submitted version. All authors contributed to the article and approved the submitted version.

## Conflict of Interest

The authors declare that the research was conducted in the absence of any commercial or financial relationships that could be construed as a potential conflict of interest.

## Publisher’s Note

All claims expressed in this article are solely those of the authors and do not necessarily represent those of their affiliated organizations, or those of the publisher, the editors and the reviewers. Any product that may be evaluated in this article, or claim that may be made by its manufacturer, is not guaranteed or endorsed by the publisher.
